# A New Operative Treatment for Hemorrhoids, with Report of a Case

**Published:** 1898-05

**Authors:** George K. Sims

**Affiliations:** Chief of Clinic, Surgical Department, University College of Medicine, Richmond, Va.


### A NEW OPERATIVE TREATMENT FOR HEMORRHOIDS, WITH REPORT OF A CASE.

By GEORGE K. SIMS, MD.,

Chief of Clinic, Surgical Department, University College of Medicine, Richmond, Va.

Read Before the Richmond Academy of Medicine and Surgery, April 26, 1898.

Having made a careful study of the various operations that have been adopted for the radical cure of hemorrhoids, I find them all subject to various objections, viz., they leave a stump that must slough off, or an open wound that must heal by granulation. This, in a location like the rectum, which can not possibly be kept aseptic for that length of time, must naturally be a slow process, and, in addition to this, there is the possibility of serious systemic poisoning should these open wounds become infected with pathogenic microbes. Again, wounds that heal under suppuration leave a great amount of cicatricial tissue to contract and distort the organs.

The operation which I propose, if properly done, will to a great extent overcome these objections. It takes more time and care and is more difficult to perform, but the advantages gained by the patient more than compensate for this additional work. Of course, it may not be applicable to every case, but with suitable modifications it may be made applicable to the majority of cases of both internal and external piles, as well as to polypi and other benign neplasms of the rectum and anus.

Operation.—The patient should have a gentle mercurial purgative on the two evenings previous to the day of operation, and a saline each morning before breakfast. This will clean out the bowels and open up the portal circulation, so that we can give the rectum a long rest afterwards. The circum-anal region should be shaved and scrubbed clean, and the rectum washed out with a large enema of warm soapsuds or carbolized water. A warm bath is given and clean linen put on. The anesthetic is now administered, after which he is placed in either the lithotomy or the Sims' position. Then introduce a speculum (Cook's and Mathews' are the best) and divulse the spincters as widely as the instrument will distend them. Then with the thumb, still further stretch until completely paralyzed. The piles will now present themselves, but not in their entirety; they should be everted as much as possible, and the rectum and circum-anal region well irrigated with a 1 to 2,000 mercuric chloride solution. The tumors, one by one, are now caught with four-pronged forceps, pulled out and held by the assistant; then, with a sharp scalpel, the mucous membrane is cut through, around the base of the pile and a silk ligature tied tightly (in the groove made by the incision), including only the blood-vessels and connective tissue. The pile is then cut off close to the ligature, leaving only enough to hold it, and the cut edges of the mucosa are brought together over the stump with continued sutures of cat-gut. If the tumor is large, with a curved needle pass a double suture through its base and ligate it in two portions, then the mucous membrane is sutured as above.

If there are external piles present, also, the same method may be used to remove them, if large and vascular, or if due to a thrombus; but if they are small or are much indurated they may be simply cut off close to the skin, any bleeding points caught with forceps and ligated, the cut edges brought into close apposition, with interrupted sutures of silk. The field of operation is again irrigated with a hot bichloride of mercury solution, the parts dusted with iodoform or aristol, the mucosa pushed in well and a small piece of iodoform gauze inserted, leaving the ends protuding from the anus. A pad of gauze is placed over the anus, and over this a pad of absorbent cotton is bound firmly with a "T" bandage. A hypodermic injection of morphia, one-quarter grain, with atropia, 1-150 grain, is given and patient put to bed.

The bowels should not be moved for three or four days, by which time the wound should be nearly healed; they should then be removed by salines ana enemata. The advantages claimed for the operation are, that by leaving only closed wounds, made under antiseptic precautions, we lessen the risk of suppuration and perhaps more serious infection; that they heal in a much shorter time and with less pain and suffering; there is less danger of hemorrhage and of distortion, and perhaps neuralgia, of the rectum, from contraction of the cicatricial tissue.

In regard to external piles I wish to emphasize the advice given by Dr. Mathews in his admirable work on the rectum: "Remove all the tumor, cutting it off close to the skin," instead of merely snipping off a small portion of it, as is advised by most authors. If small ones are left they are apt to become inflamed, and they, as well as the stumps left, tend to get much larger, often necessitating another operation to remove them.

The following case, which was a very severe and complicated one, will illustrate the success of the operation, although it was done under very unfavorable circumstances and surroundings:

W. C., aged thirty, had been suffering very much for some months with pains in the region of the anus and surrounding parts, especially during and after stools. They had got so severe that he had to take his bed, and could get no relief from the many remedies and treatment he had received. Upon examination I found a large and inflamed anal fissure, and about two-third of the circumference of the anus was encircled by very large, indurated and ulcerated hemorrhoids. Owing to these conditions I did not examine the interior of the rectum, but advised an operation as the only means of getting relieved. To this he consented, but, being opposed to the hospital, as many are, I decide to operate at his honse. After being prepared as above, chloroform was administered by Dr. Charles

M. Edwards, he being my only assistant, except a man to hold his limbs out of the way. He was placed in the Sims' position, the sphincters thoroughly paralyzed by stretching, the mucous membrane everted, and the parts washed cleaned with a warm antiseptic solution. This revealed the presence of two medium-sized internal piles, which were caught with forceps and pulled out. The mucosa was cut through around the base of the pedicle and a silk ligature tied tightly in the groove made by this incision. The tumor was then cut off close to the ligature and the cut edges of the mucous membrane were brought into close apposition with a continuous suture of cat-gut, covering over the stump. The external piles being of the fibrous, indurated variety, were simply trimmed off close to the skin without being clamped; several small arteries were ligated and the cut edges brought together with interrupted sutures of silk. The parts were then sponged off with hot mercuric chloride solution and a piece of iodoform gauze inserted into the anus with the end protruding. Another piece of gauze was then placed over the anus, covered by a pad of absorbent gauze, and the patient put to bed. A hypodermic of morphine and atropine was given to relieve the pain. This was not repeated. The bowels were moved on the fourth day by salines and enemata. He was out of the bed in less than a week, and on the tenth day came to my office and I removed the stitches. All the wounds were healed nicely and he was feeling very well. He returned to his work several days later, being completely cured, and has had no return of the trouble since.

---

### SPRING.

All nature wakes, spring flowers blow,
Thoughts turn to Aphrodite,
And balmy south-winds whisper low,
"Hydrarg chloridum mite."

